# Depression in HIV-positive women is associated with changes in antiretroviral treatment regimens

**DOI:** 10.7448/IAS.17.4.19720

**Published:** 2014-11-02

**Authors:** Claus Philippe Küpper-Tetzel, Siri Göpel, Pavel Khaykin, Timo Wolf, Christoph Stephan, Eva Herrmann, Hans-Reinhard Brodt, Annette Haberl

**Affiliations:** 1 Department of Infectious Diseases, Medical Clinic II, University Clinic Frankfurt, Frankfurt, Germany; 2 Department of Biostatistics and Mathematic Modelling, University Clinic Frankfurt, Frankfurt, Germany

## Abstract

**Introduction:**

Depression is a co-morbidity of clinical significance in HIV-positive patients with an estimated prevalence of more than 20%. Sex and gender-related differences in depression are well described in HIV-negative populations, demonstrating that more women are being affected. So far little is known about frequency and characteristics of depression in HIV-positive men and women.

**Materials and Methods:**

Primary objective of our prospective epidemiological study was the evaluation of the Beck score for depression in male and female patients of the Frankfurt HIV Cohort. The Beck Depression Inventory (BDI-II) is a self-report symptom inventory made up of 21 questions, each with 4 possible answers, correlating with a certain point value. Interpretation: score 14–19: mild depression; score 20–28: moderate depression; score ≥29: severe depression. Secondary objectives of the analysis were factors that might possibly influence the disposition for depression in HIV-positive patients, e.g. age, antiretroviral treatment history, co-morbidities and socioeconomic status.

**Results:**

Between January and October 2013, 348 patients were enrolled in the study, 161 women and 187 men of the Frankfurt HIV Cohort, who had a routine appointment at the HIV-Center of the University Clinic Frankfurt. The mean age of all study participants was 45 years (range 22–80). The majority of patients were on antiretroviral therapy (91%) at study entrance. The median BDI-II score in all patients was 8 (0–49); in female patients 10 (0–42), in male patients 6 (0–49), respectively (Table 1). Significant more women than men showed a score for moderate depression (p=0.006). Factors associated with a BDI-II score ≥20 in women were older age (>45 years), living alone, unemployment and the number of prior changes in antiretroviral therapy.

**Conclusions:**

Depression in people living with HIV shows sex and gender-related differences that might also influence antiretroviral treatment strategies. HIV specialists should be aware of these gender-specific aspects and consider routine screening for depression especially in female patients of older age or those with multiple therapy changes in history.

**Table 1:** Distribution of BDI-II scores in men and women

	All patients n=348	Men n=187	Women n=161
BDI-II score 1–19 n (%)	288 (82%)	165 (88%)	123 (76%)
BDI-II score 20–28 n (%)	41 (12%)	13 (7%)	28 (18%)
BDI-II score ≥29 n (%)	19 (6%)	9 (5%)	10 (6%)

